# How can college teachers’ information literacy empower their innovative teaching behaviors under the background of informatization?

**DOI:** 10.1371/journal.pone.0294593

**Published:** 2023-12-15

**Authors:** Lianghua Gong

**Affiliations:** School of Modern Economics and Management, Jiangxi University of Finance and Economics, Gongqing city, China; BRAC Business School, BRAC University, BANGLADESH

## Abstract

The innovative teaching behaviors of college teacher are of positive significance for promoting the construction of educational informatization in China and realizing the high-quality development of higher education. Based on the theory of social cognition and theory of behavioral change, the paper explores the influential mechanism and its boundary conditions of college teachers’ information literacy on their innovative teaching behaviors during the new era of educational informatization. Through the investigation of 280 college teachers, it is found that: (1) information literacy and its dimensions of information knowledge and information capability have a significant positive effect on innovative teaching behavior; (2) Creative self-efficacy plays a mediating role between information literacy (information knowledge and information capability) and innovative teaching behavior; (3) College innovation climate significantly moderates the positive relationship between information literacy (information awareness and information knowledge) and creative self-efficacy; (4) College innovation climate significantly moderates the mediating effect of creative self-efficacy between information literacy (information awareness and information knowledge) and innovative teaching behavior. Therefore, colleges and universities should focus on enhancing teachers’ information literacy level and their creative self-efficacy, creating an excellent atmosphere for innovation, and encouraging college teachers to actively carry out their innovative teaching practice in the new stage of educational informatization.

## 1 Introduction

Since Chinese Education Ministry put forward the Ten-Year Development Plan of Education Informatization in 2012, China’s education informatization construction has taken a new step after ten years of layout and development. At the new stage of comprehensive development of educational informatization, the deep integration of new information technology and course teaching by college teachers to carry out teaching innovation is not only beneficial to colleges and universities for further improving the quality and effectiveness of course teaching [1], but also helps colleges and universities to cultivate innovative talents and thus achieve high-quality and modernized development of higher education [2]. Therefore, the university teachers’ innovative teaching behavior integrating information technology in the new stage of education informatization and its influential factors deserve to be paid high attention.

Scholars have explained the causes of teachers’ innovative teaching behavior at the individual level such as proactive personality [3], technological innovation acceptance [4], teaching self-efficacy [5] or environmental level such as school culture [6], leadership style [7,8], but researches that combine various factors to study college teachers’ information-based innovative teaching behaviors are relatively scarce. Modern information technology has reshaped the form, process and the content of teaching innovation in college courses, and information literacy of college teachers not only has a positive effect on promoting the use of new forms and contents and improving teaching effectiveness in the process of informatization teaching, but also has a positive impact on promoting teachers’ professional growth and making college teaching and education quickly meet the real needs of the era change of education informatization and the development of modern intelligent society [1]. Therefore, the information literacy level of college teachers is important for them to carry out innovative teaching activities and thus promote the sustainable development of higher education informatization in China. Although some studies have focused on the influence of teachers’ information literacy on innovative teaching behavior [1,9], however, these studies took information literacy as a single dimensional variable to investigate its effects. According to the “Information Literacy Framework for Higher Education” issued by the Council of American Association of University and Research Libraries (ACRL) and previous studies, college teachers’ information literacy has rich connotations and conceptual dimensions. Then, how do different dimensions of information literacy affect teachers’ innovative teaching behavior? And are there any differences existing in the influence of different dimensions on innovative teaching behavior?

According to social cognitive theory, human activities are determined by the interaction of three factors: individual behavior, individual cognition and other individual characteristics, and the external environment in which individuals live [10], so college teachers’ innovation cognition (for example creative self-efficacy) is an important factor that may affect college teachers’ innovative teaching behavior. In the new stage of the rapid development of educational informatization, college teachers will inevitably encounter cognitive and operational obstacles of new technology to integrate new information technology to carry out teaching innovation. Therefore, only when they believe that they have the ability to overcome the difficulties and can achieve the desired innovation goals (high self-efficacy), will they be willing to continue. As Bandura puts it, “Innovation requires an unshakeable sense of efficacy and a long-term bet of time and effort” [11]. Therefore, college teachers’ creative self-efficacy has a positive significance on their innovative teaching behavior [12,13]. But what is the effect of teachers’ creative self-efficacy between their information literacy and innovative teaching behavior? For this issue, the current relevant research is not clear.

Besides individual factors, environmental factors such as college innovation climate are also important factors affecting teaching innovation [14,15]. This is because school innovation climate, for example, the managements’ support for innovative teaching and the training and re-education provided, can not only stimulate teachers to bring more diversified teaching methods, but also enhance teachers’ self-efficacy in using new teaching tools [15,16]. However, most current studies mainly explored the direct effects and mediating effects of school innovation climate, ignoring the effects of interaction between innovation climate and other factors on innovative teaching behavior. According to the social cognitive theory, the interaction between environmental factors and personal characteristics may affect individual cognition and thus their behaviors. Then, does the interaction between college innovation climate and teachers’ information literacy (and its dimensions) affect their creative self-efficacy? And how does the interaction further affect the action mechanism of information literacy on innovative teaching behavior?

Therefore, this study takes creative self-efficacy as the mediator and college innovation climate as the moderator to explore the mechanism and boundary conditions of college teachers’ information literacy on their innovative teaching behavior under the conditions of informatization, so as to achieve three research objectives: (1) examining the effects of information literacy and its dimensions on college teachers’ innovative teaching behavior under the condition of informatization; (2) clarifying the mechanism of college teachers’ information literacy affecting innovative teaching behavior, that is, investigating the mediating role of creative self-efficacy between information literacy and innovative teaching behavior; (3) exploring the moderating role of college innovation climate on information literacy and creative self-efficacy, and thus examining the moderated mediating effect of college innovation climate on the above intermediary mechanism.

## 2 Literature and hypothesis

### 2.1 Information literacy as a predictor of innovative teaching behavior of college teachers

Information literacy of college teachers is a comprehensive quality of college teachers to use information and information technology purposefully and reasonably [17], which specifically includes dimensions of information awareness, information knowledge and information capability. Information awareness refers to the individual’s sensitivity to information, which is reflected in the conscious awareness of information value and keen judgment and analysis. Information knowledge is the sum of people’s knowledge and experience accumulated in using information technology tools, expanding information dissemination channels and improving information exchange efficiency. Information capability is people’s ability to use information knowledge and conduct information activities [18]. Under the background of informatization, information literacy is an important factor that determines individual innovation intention, behavior and innovation performance. Employees’ information literacy is conducive to improving their perceived ease of use of digital technologies, which in turn affects their attitude and willingness to use them [19]. Information-related skills, aptitudes and resources possessed by employees can help them acquire new ideas and solutions to problems, thus developing their innovative work behavior [20]. In education field, teachers’ information literacy not only has a positive impact on their information technology integrated instructions, but also shows notably positive effect on teaching effectiveness [1].

Under the background of the rapid development of information technology, teachers should not only pay attention to the upcoming information technology related to teaching and the resulting constantly updated information knowledge, but also integrate these technologies and knowledge deeply into their own teaching process. Therefore, the study draws on the views of Chinese scholars and refines innovative teaching behavior as a teaching practice in which teachers constantly adopt new teaching concepts, update teaching content, and apply new teaching methods and means to improve teaching effect with the goal of better growth and development of students in the teaching process [13]. Innovative behaviors are critical to any organization, no exception for education field. This is because innovative teaching not only can help attract and sustain students’ interest, encourage their academic engagement and promote their self-efficacy beliefs [21], but also can be benefit for university teachers adapting to new technologies and insights related to teaching constantly, improving teaching effectiveness, meeting the rapidly changing needs of talent training, and promoting the development of education profession, school organization and knowledge society [1,22].

As for the effect of college teachers’ information literacy on innovative teaching behavior, behavioral change theory proposes that information and knowledge are the basis and key factors for individual behavioral decision-making [23]. Therefore, this study holds that college teachers’ information literacy plays a decisive role in their innovative teaching behaviors that integrate information technology. A high level of information literacy can effectively reduce teachers’ pressure of using information technology [24], which directly affects the use of information technology. On the contrary, if college teachers lack information literacy, they will not be able to cope with the challenges brought by new teaching methods and means [25], so the behavioral tendency of integrating information technology into teaching to carry out teaching innovation will be weak. One research supported the positive relationship between information literacy and teachers’ innovative behavior [9].

In terms of specific dimensions, college teachers with high information awareness can effectively solve the negative impact brought by technology pressure [26], have a more sensitive discovery of information about new technologies under the background of informatization and a more comprehensive cognition of the value of such information, thus they will carry out more conscious information behavior [18]. Knowledge and skills related to modern information technology can not only effectively reduce teachers’ computer anxiety, but also directly affect the use of these technologies by college teachers [27]. Therefore, information knowledge and information capability are the prerequisites for college teachers to implement innovative teaching behaviors in the information age [24]. In sum, this study proposes the following hypothesis:

H1: College teachers’ information literacy has a positive effect on their innovative teaching behavior;
H1a-1c: College teachers’ information awareness, information knowledge and information capability have a positive effect on their innovative teaching behavior.

### 2.2 Creative self-efficacy as a mediator

Creative self-efficacy refers to the belief in one’s own capability to perform an innovative task effectively and attain certain desired goals [28]. According the definition, teachers with high creative self-efficacy believe that they not only have the knowledge and ability to carry out teaching innovation activities, but also can obtain positive innovation results through their actions and efforts, so they will have more motivations to carry out teaching innovation [29]. Therefore, teachers’ creative self-efficacy has positive significance to their teaching innovation [12,13]. Besides, according to behavioral change theory, individual behavior is not only influenced by information or knowledge obtained from the outside world, but also indirectly influenced by his subjective perception [23]. Among various subjective perception factors, creative self-efficacy is an important cognition factor that is close to their innovative teaching behavior, so we hold that creative self-efficacy is also an important mediator that the effect of college teachers’ information literacy is transmitted to innovative teaching behavior. In other words, the higher the information literacy level of college teachers, the stronger their consciousness and ability to capture and use such information and information technology, thus they more believe that they can integrate new information technology into teaching innovation and achieve certain desired teaching innovation results, so they will carry out more innovative teaching activities in the new era [5,12].

Specifically, information awareness can help college teachers judge whether the strategies and methods of information collection are appropriate and whether the information found can meet the needs of teaching innovation. Information knowledge can help identify the new technologies that can be used in teaching innovation, while information capability can help integrate new information, new technology into teaching process required for teaching innovation effectively. All these qualities can significantly increase the successful experience and self-confidence of college teachers in carrying out teaching innovation, and then stimulate them to carry out more teaching innovation. In this regard, Chinese scholars also found that the sense of creative self-efficacy plays a partial mediating role between the teaching innovation ability and college teachers’ innovative teaching behavior [13]. In sum, the study proposes the following hypothesis:

H2: College teachers’ creative self-efficacy plays a mediating role between their information literacy and innovative teaching behavior;
H2a-2c: College teachers’ creative self-efficacy plays a mediating role between their information awareness, information knowledge, information capability and innovative teaching behavior.

### 2.3 College innovation climate as a moderator

Innovation climate in colleges and universities is the perception of college teachers on the innovation environment of their school, such as sufficiency of teaching resources, encouragement from leaders and administrative support level [30]. As mentioned above, teachers’ teaching innovation will face many obstacles and challenges, so school innovation support and encouragement are the external forces for them to overcome difficulties and continue to move forward. In this regard, some scholars proposed that the school innovation atmosphere is conductive to stimulating teachers’ creative teaching [14,15]. According to social cognitive theory, the interaction between college innovation climate (environmental factors) and teachers’ information literacy (personal characteristics) will affect college teachers’ creative self-efficacy (innovation cognition). Therefore, the study holds that college innovation climate may moderate the relationship between their information literacy (and its various dimensions) and creative self-efficacy.

When the innovation climate is strong in colleges and universities, the support and encouragement from teaching teams or leaders and the relaxed climate created by them will make teachers with weak information awareness still have confidence to carry out innovative teaching activities. A perfect further education and training mechanism can effectively reduce the difficulty of college teachers lacking information knowledge to carry out teaching innovation. Adequate support for teaching innovation, such as continuous support of teaching resources and administrative personnel, can enhance the confidence of teachers with weak information capability to achieve the expected innovation results [31]. Therefore, even if college teachers’ information awareness, knowledge and ability are not high, that is, the overall level of information literacy is not high, they still have a high level of creative self-efficacy, which means high innovation climate will weaken the positive effect of information literacy and its various dimensions on creative self-efficacy. On the contrary, in colleges with poor innovation climate and lack of resources, systems and environmental supports, teachers’ creative self-efficacy is mainly derived from their own information awareness, information knowledge and ability, that is, mainly depends on their own information literacy, so that the positive impact of information literacy and its various dimensions on creative self-efficacy will be enhanced. In sum, the study proposes the following hypothesis:

H3: College innovation climate can be a moderator that weakens the positive relationship between teachers’ information literacy and their creative self-efficacy;
H3a-3c: College innovation climate can be a moderator that weakens the positive relationship between teachers’ information awareness, information knowledge, information capability and their creative self-efficacy.

Implementing teaching innovation with information technology is actually a kind of teaching activity to explore the unknown, of which the behavior results have certain uncertainties and external dependence. Therefore, the innovative teaching behavior of college teachers will not only be affected by their personal factors, but also vary due to different external environments. Some scholars have proposed that the innovation climate in colleges and universities is the premise and guarantee for teachers’ innovative teaching behaviors, and plays a positive role in the possibility and quality of teachers’ innovative teaching activities [32]. This positive effect can be influenced by the teachers’ psychological process [33], that is, college teachers may adjust their self-cognition according to the information transmitted by the environment and thus determine their innovative teaching behavior.

As mentioned above, when colleges and universities have a good innovation climate, even if teachers’ information awareness, information knowledge and information capability are not high, that is, the overall level of information literacy is not high, the high creative self-efficacy brought about by high-level innovation support will still stimulate their deeper participation in creative teaching activities. That means, high innovation climate will weaken the mediating effect of creative self-efficacy between information literacy (and its dimensions) and innovative teaching behavior. On the contrary, when the innovation climate in colleges and universities is poor, teachers perceive that the external environment is not conducive to teaching innovation, and they can only build up the confidence to carry out teaching innovation through their information awareness, information knowledge and capability, that is, relying on their own information literacy to bring about innovative teaching behavior. That means, low innovation climate will strengthen the mediating effect of creative self-efficacy between information literacy (and its dimensions) and innovative teaching behavior. In sum, the study proposes the following hypothesis:

H4: College innovation climate can moderate the mediating effect of teachers’ creative self-efficacy between their information literacy and innovative teaching behavior;
H4a-4c: College innovation climate can moderate the mediating effect of teachers’ creative self-efficacy between their information awareness, information knowledge, information capability and innovative teaching behavior.

**Fig 1:**
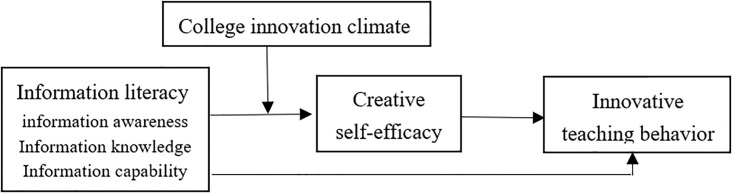
Conceptual model diagram.

The conceptual model constructed in this study is shown in Fig 1.

## 3 Material and methods

### 3.1 Study design and participants

The researcher conducted a retrospective questionnaire survey on full-time college teachers to empirically examine the mechanism of information literacy of college teachers on their innovative teaching behavior in the context of informatization. According to the research purpose, research context and previous research results, the formal questionnaire of the study was formed. The whole formal questionnaire included four parts: the basic information of teachers, the measurement of information literacy level, some aspects of teaching innovation, college innovation climate and future prospects, with a total of 48 questions. There were 8 questions in basic information part, including gender, age, education, professional title, teaching school, etc.; the measurement of teachers’ information literacy level had 19 items; and the third and fourth part included 10 and 11 items respectively. After the content of the questionnaire was determined, the researcher produced an electronic questionnaire on the well-known online survey platform “Questionnaire Star” in China (link address is https://www.wjx.cn/vm/tUUmRZW.aspx#).

In order to receive as many samples as possible, the study selected colleges and universities in the province and nearby provinces for data collection. From August 20 to September 20, 2022, the research recruited participants through QQ and WeChat groups for various university teachers’ work communication. After expressing interest in participating, college teachers were sent an e-mail with an electronic informed consent form and a questionnaire website. They were asked to sign the informed consent form, return it by mail, and complete the questionnaire from the website. We also sampled the teachers’ education background, professional titles and types of teaching schools in different universities by stratified quotas to ensure the representativeness of the samples when issuing questionnaires.

A total of 367 questionnaires were sent out in the formal investigation, and 325 were recovered, with a recovery rate of 88.55%. After eliminating questionnaires with repeated submissions, large blank choices and a large number of identical item choices, a total of 280 effective questionnaires were obtained, with an effective rate of 86.15%. Among the valid samples, 76 (27.14%) has a doctor’s degree and 171 (61.07%) has a master’s degree. There were 89 people (31.78%) with associate professor title and above, and 144 people (51.43%) with lecturer title. There are 113 people (40.36%) from the first batch of ordinary undergraduate group (including 985 and 211), and 142 people (50.71%) from the second batch of ordinary undergraduate Group (See Table 1).

**Table 1:** Distribution of teachers’ background.

Participant demographics	Categories	Frequency	%
Gender	male	111	39.64
	female	169	60.36
Age	≤30	51	18.21
	31–40	139	49.65
	41–50	65	23.21
	>50	25	8.93
Education	Bachelor	33	11.79
	Master	171	61.07
	Doctor	76	27.14
Professional title	Teaching assistant	36	12.86
	Lecturer	144	51.43
	Associate professor	68	24.28
	professor	21	7.5
	others	11	3.93
School type	985\211	25	8.93
	first batch of ordinary undergraduate	88	31.43
	second batch of ordinary undergraduate	142	50.71
	Higher vocational college	25	8.93

### 3.2 Variables and measurements

All the research tools used in this study were from domestic and foreign maturity scales, and a strict “translation-back translation” process was adopted. Two professors engaged in pedagogical research were invited to review the final translation, and a formal questionnaire was formed after a small range of modifications. Except for the 7-point Likert scale used for creative self-efficacy, the other measuring tools used 5-point scale.

1. Information literacy. The study referred to Chinese scholar Wang to divide college teachers’ information literacy into three dimensions: information awareness, information knowledge and information capability [34]. For the measurement of the three dimensions, we adapted the scale of college teachers’ information literacy used by Su and Wang [35]. There are 19 items in the scale, among which information awareness includes 6 items such as “You can realize that information literacy is very important for teaching work”; information knowledge includes 5 items such as “You can master the professional knowledge of information technology and the subject you teach”; information capability includes eight items such as “You can efficiently retrieve information useful for course teaching”. The results of confirmatory factor analysis showed that the data fit the model of three factors and one higher-order factor. The Cronbach’s α of information literacy is 0.945, and the Cronbach’s α of the three dimensions is 0.898, 0.856, and 0.907 respectively.
2. Innovative teaching behavior. Scott and Bruce’s innovative teaching behavior scale was adopted to measure the innovative teaching behavior tendency of college teachers [36]. The scale has six items, including representative items such as “You often propose new ideas for integrating technology and teaching”. The scale has good reliability and validity, which is widely used in the researches of teachers’ innovative teaching behavior in various colleges and universities at home and abroad. Cronbach’s α of the scale in this sample is 0.887.
3. Creative self-efficacy. The Chinese version of creative self-efficacy scale was adopted to measure the sense of college teachers’ creative teaching self-efficacy [3], which was also proved to have good convergence, differentiation and predictive validity [37]. The scale has 4 items, including representative items such as “You can creatively solve difficult problems in teaching”. Cronbach’s α of the scale in this sample was 0.916.
4. College innovation climate. The study adopted the organizational innovation climate Scale adapted by Yu to measure the teaching innovation climate in colleges and universities [30]. The scale has a total of 8 items, including representative items such as “Your school welcomes innovative teaching ideas”. The scale also has good reliability and validity, and is widely used in the research of innovative behavior at home and abroad. Cronbach’s α of college innovation atmosphere in this sample is 0.907.
5. Control variables. Referring to previous studies, this study takes the gender, age, education and school type as control variables.

### 3.3 Analysis tools and methods

Statistical software SPSS22.0 and AMOS23.0 were used to test the reliability and validity of all measuring tools, common method variance and the fit of the model. Then, the researcher used SPSS22.0 to calculate the Pearson correlation coefficient to examine the relationship between all the studied variables. After that, AMOS23.0 was used to examine the direct effect of information literacy (and its dimensions) on innovative teaching behavior and the mediating effect of creative self-efficacy. For the test of mediating effect, the number of repeated samplings was set to 5000 and the confidence interval was 95%. To examine the moderation effect of college innovation climate between information literacy (and its dimensions) and creative self-efficacy, the researcher run a series of linear regressions by SPSS22.0 to predict the effects of information literacy (and three dimensions), college innovation climate and their interaction on creative self-efficacy sequentially. Finally, model 7 of Process V3.4 macro program in SPSS22.0 was selected to test the moderated mediation effect of college innovation climate.

## 4 Results

### 4.1 Common method variance

We conducted the Harman’s single factor test to check the common method bias due to the self-reported items. Exploratory factor analysis was performed for 37 items of all variables. The results showed that 6 factors had eigenvalues greater than 1, explaining 67.59% of the variation. The variance explained by the first factor is 38.91%, which is lower than 40% of the total explained amount, indicating that the common method bias in the study is acceptable.

### 4.2 Confirmatory factor analysis

Due to the large number of variable items, the scales of three dimensions of information literacy and college innovation climate are packaged respectively before confirmatory factor analysis. The packaged scale of information literacy and college innovation climate contains 10 and 4 items respectively. Then it is analyzed together with creative self-efficacy and innovative teaching behavior. The results of confirmatory factor analysis are shown in Table 2. The fit of the six-factor model is the best and significantly superior to other competitive models, indicating that all variables in the study have a good differentiation degree.

**Table 2:** Results of confirmatory factor analysis.

Models	x^2^	df	x^2^/df	RMSEA	CFI	TLI	IFI
Six-factor model (IA; IK; IC; CSE; CIC; ITB)	566.056	241	2.349	0.070	0.939	0.930	0.940
Four-factor model (IL; CSE; CIC; ITB)	722.432	248	3.034	0.084	0.905	0.894	0.906
Three-factor model (IL; CSE+CIC; ITB)	1245.634	249	5.003	0.120	0.812	0.792	0.813
Two-factor model (IL+CSE+CIC; ITB)	1601.298	251	6.380	0.139	0.746	0.721	0.747
Single factor model (IL+CSE+CIC+ITB)	1718.048	252	6.818	0.144	0.724	0.698	0.725

Note: IA, IK, IC, IL, CSE, CIC and ITB in this study represent information awareness, information knowledge, information capability, information literacy, creative self-efficacy, college innovation climate, innovative teaching behavior respectively.

### 4.3 Descriptive statistics and correlation analysis

Table 3 shows descriptive statistical analysis of all variables and correlation coefficients. As shown in Table 3, college teachers’ information literacy and its three dimensions are significantly positively correlated with their creative self-efficacy and innovative teaching behavior, and creative self-efficacy is also significantly positively correlated with innovative teaching behavior. These results are in line with the basic expectation and preliminarily support the research hypothesis.

**Table 3:** Descriptive statistical analysis and correlation coefficient.

Variables	Mean	SD	1	2	3	4	5	6	7
1 Information literacy	3.79	0.61	1						
2 Information awareness	4.03	0.71	0.846**	1					
3 Information knowledge	3.56	0.69	0.818**	0.616**	1				
4 Information capability	3.74	0.67	0.863**	0.649**	0.799**	1			
5 Creative self-efficacy	5.28	0.96	0.696**	0.523**	0.631**	0.697**	1		
6 College innovation climate	3.81	0.66	0.524**	0.436**	0.459**	0.497**	0.537**	1	
7 Innovative teaching behavior	3.68	0.66	0.667**	0.580**	0.688**	0.659**	0.646**	0.484**	1

Note:

* p < 0.05,

** p < 0.01,

*** p < 0.001.

### 4.4 Hypothesis testing

1. Main effect. As shown in Table 4, after controlling variables such as gender and age, the standardization coefficient of information literacy affecting innovative teaching behavior is 0.54, and the significance of the two-tail test is less than 0.001, so it is determined that information literacy has a significant positive effect on innovative teaching behavior, that is, H1 is supported. Similarly, the dimensions of information literacy such as information knowledge (β = 0.17, p = 0.009 < 0.01) and information capability (β = 0.30, p = 0.000 < 0.001) also have significant positive effects on innovative teaching behavior, so H1b and H1c are also supported. The positive effect of information awareness on innovative teaching behavior (β = 0.05, p = 0.25 > 0.05) was not significant, so H1a was not supported. It is worth noting that among the three dimensions of information literacy, the impact of information capability on innovative teaching behavior is the greatest.

**Table 4:** Path analysis results.

Model pathways	β	P	95% confidence interval
IL → ITB	0.541	0.000	[0.416, 0.664]
IA → ITB	0.053	0.250	[-0.037, 0.148]
IK → ITB	0.167	0.009	[0.041, 0.290]
IC → ITB	0.301	0.000	[0.164, 0.435]
IL → CSE → ITB	0.307	0.000	[0.204, 0.412]
IA → CSE → ITB	0.036	0.120	[-0.008, 0.084]
IK → CSE → ITB	0.079	0.013	[0.017, 0.144]
IC → CSE → ITB	0.193	0.000	[0.122, 0.275]

Note: β is the standardized coefficient.

2. Mediation effect. The test results of the mediation effect show (see Table 4) that the indirect effect of information literacy on innovative teaching behavior through creative self-efficacy is 0.31, and the 95% confidence interval ([0.204, 0.412]) does not contain 0, indicating that the mediating effect of creative self-efficacy between information literacy and innovative teaching behavior is significant, so H2 is supported. As for three dimensions, the indirect effect of information awareness on innovative teaching behavior through creative self-efficacy is 0.04, and the 95% confidence interval ([-0.008, 0.084]) contains 0, indicating that the mediating effect of creative self-efficacy between information awareness and innovative teaching behavior is not significant, so H2a is not verified. The indirect effects of information knowledge and information capability on innovative teaching behavior through creative self-efficacy are 0.08 and 0.19 respectively, 95% confidence intervals (that is [0.017, 0.144], [0.122, 0.275] respectively) do not include 0. It means that creative self-efficacy has a significant mediating effect between information knowledge or information capability and innovative teaching behavior, so H2b and H2c are supported.

3. Moderation effect. In order to avoid the multicollinearity problem, the variables involved were first decentralized before the hierarchical regression analysis was used to test the moderating effect of college innovation climate. The results of hierarchical regression are shown in Table 5. The interaction between information literacy and college innovation climate significantly negatively affects creative self-efficacy (β = -0.10, p < 0.05), indicating that college innovation climate negatively moderates the relationship between information literacy and teachers’ creative self-efficacy, that is, H3 is established.

**Table 5:** The moderating effect of college innovation climate.

Variables	Creative self-efficacy								
	M1	M2	M3	M4	M5	M6	M7	M8	M9
Gender	-0.027	-0.024	-0.034	-0.036	-0.047	0.001	-0.006	-0.041	-0.048
Age	-0.011	0.007	0.005	-0.005	-0.015	0.008	0.011	0.019	0.019
Education	-0.035	-0.030	-0.037	-0.014	-0.015	-0.013	-0.027	-0.014	-0.019
Teaching school	0.039	0.019	0.036	0.054	-0.078	-0.004	0.013	-0.012	-0.003
IL	0.698***	0.577***	0.568***						
IA				0.364***	0.323***				
IK						0.489***	0.501***		
IC								0.574***	0.571***
CIC		0.234***	0.220***	0.372***	0.362***	0.314***	0.297***	0.254***	0.243***
IL*CIC			-0.104*						
IA*CIC					-0.155**				
IK*CIC							-0.118**		
IC*CIC									-0.064
R^2^	0.490	0.529	0.539	0.395	0.416	0.475	0.489	0.536	0.540
F	52.592***	51.075***	45.429***	29.752***	27.734***	41.243***	37.139***	52.532***	45.549***

As for the specific dimension, the interaction of information awareness and college innovation climate significantly negatively affect creative self-efficacy (β = -0.16, p < 0.01), and the interaction of information knowledge and college innovation climate also significantly negatively affect creative self-efficacy (β = -0.12, p < 0.01). It shows that the college innovation climate has a significant moderating effect on the relationship between teachers’ information awareness, information knowledge and creative self-efficacy, so H3a and H3b are supported. However, the interaction between information capability and college innovation climate has no significant negative effect on creative self-efficacy (β = -0.06, p > 0.05), indicating that the moderating effect of college innovation climate on the relationship between information capability and innovation self-efficacy is not significant, that is, H3c is not supported.

**Fig 2:**
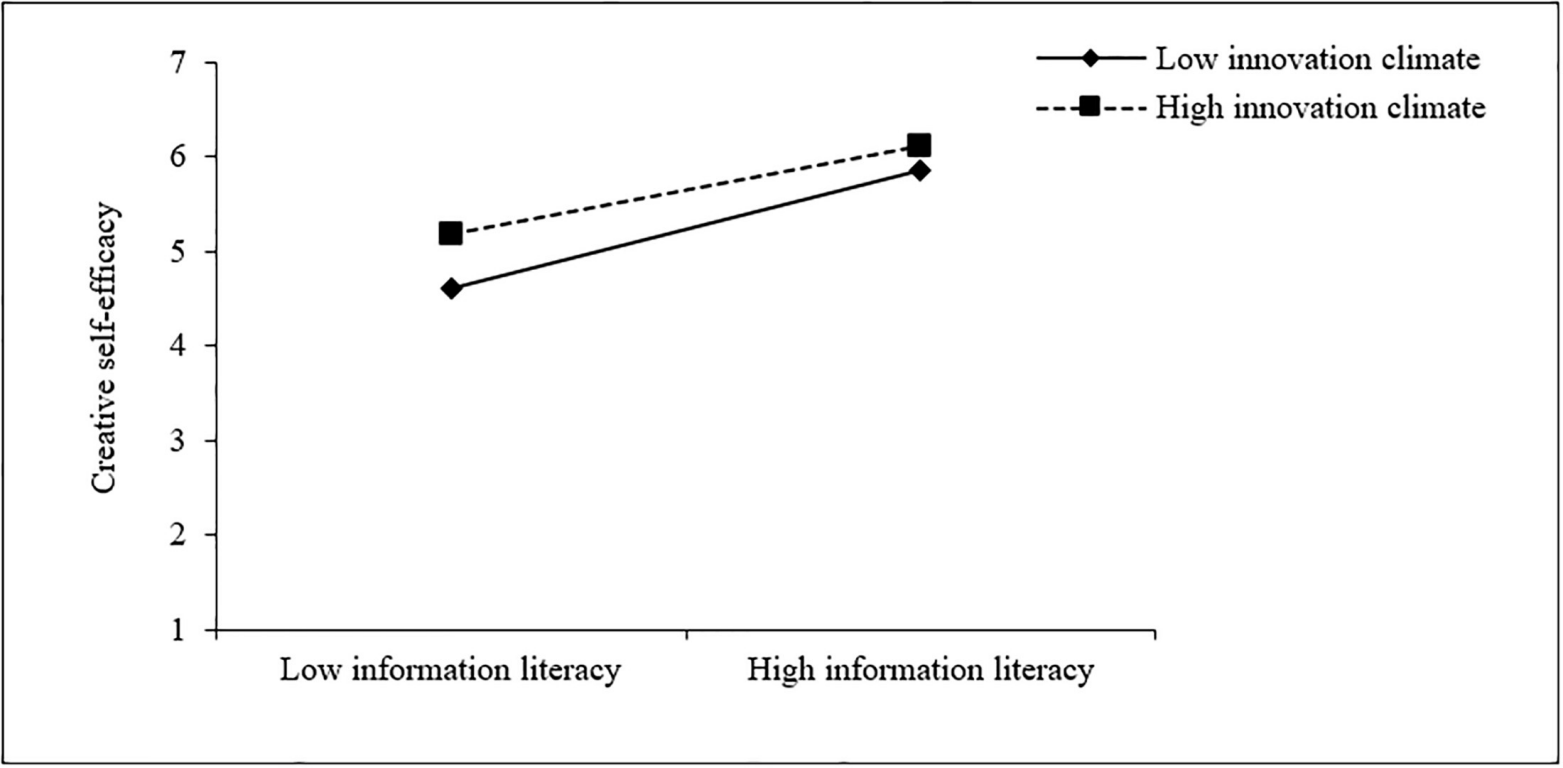
The moderating effect of college innovation climate on the relationship between teachers’ information literacy and creative self-efficacy.

So as to have a more intuitive understanding of the moderating effect of college innovation climate between teachers’ information literacy, its dimensions and creative self-efficacy, this study further divided college innovation climate into high and low groups according to the principle of one standard deviation below and one standard deviation above the mean, and drew the moderating effect diagram. As shown in Fig 2, for teachers with low college innovation climate (M-1SD), the positive effect of information literacy on creative self-efficacy (simple slope = 1.02, t = 11.11, p < 0.001) is slightly stronger than that of teachers with higher innovation climate (M+1SD) (simple slope = 0.76, t = 7.65, p < 0.001). This means that when the college innovation climate is poor, teachers’ information literacy plays a stronger role in their creative self-efficacy, which further supports H3.

**Fig 3:**
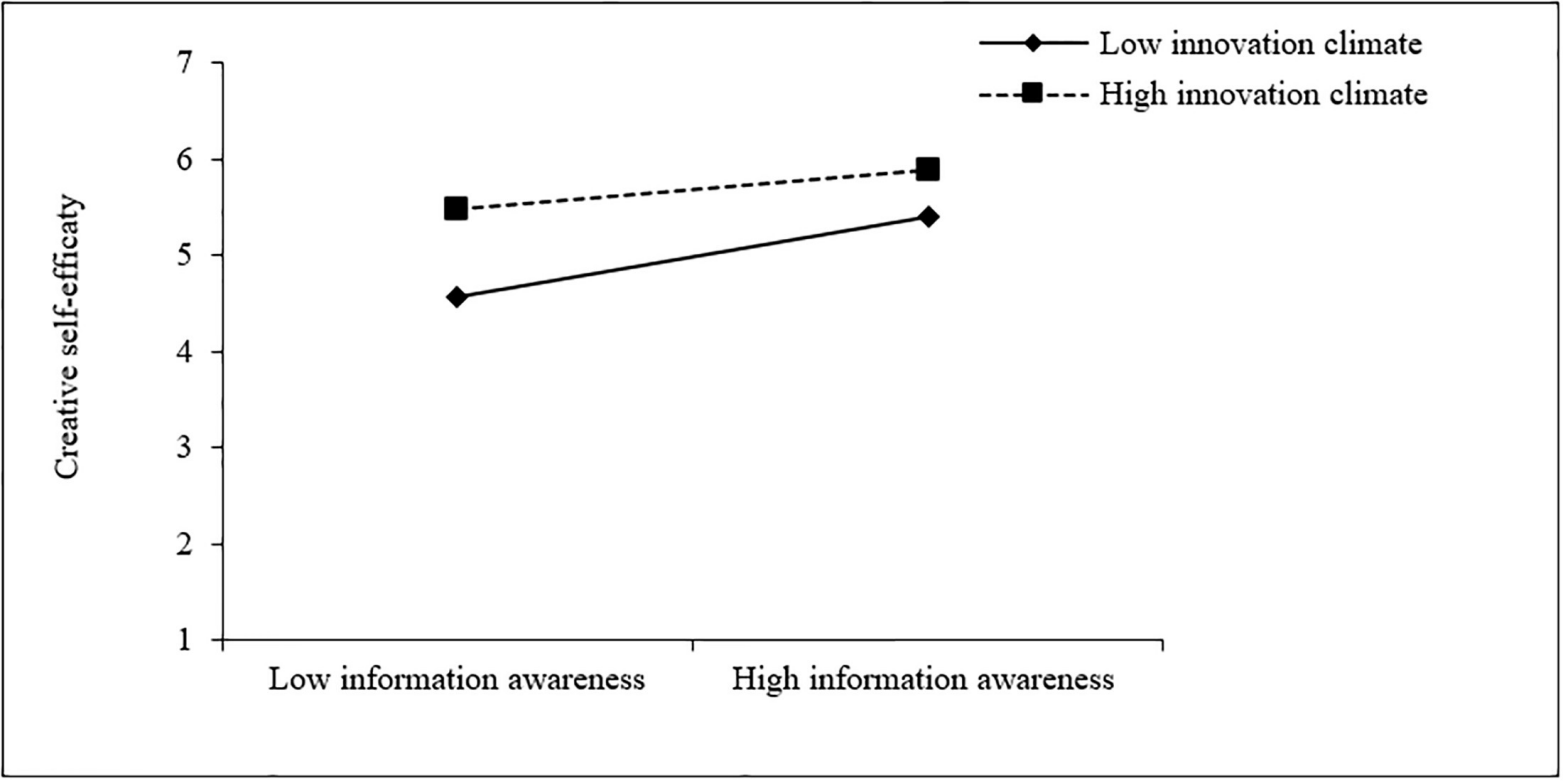
The moderating effect of college innovation climate on the relationship between teachers’ information awareness and creative self-efficacy.

**Fig 4:**
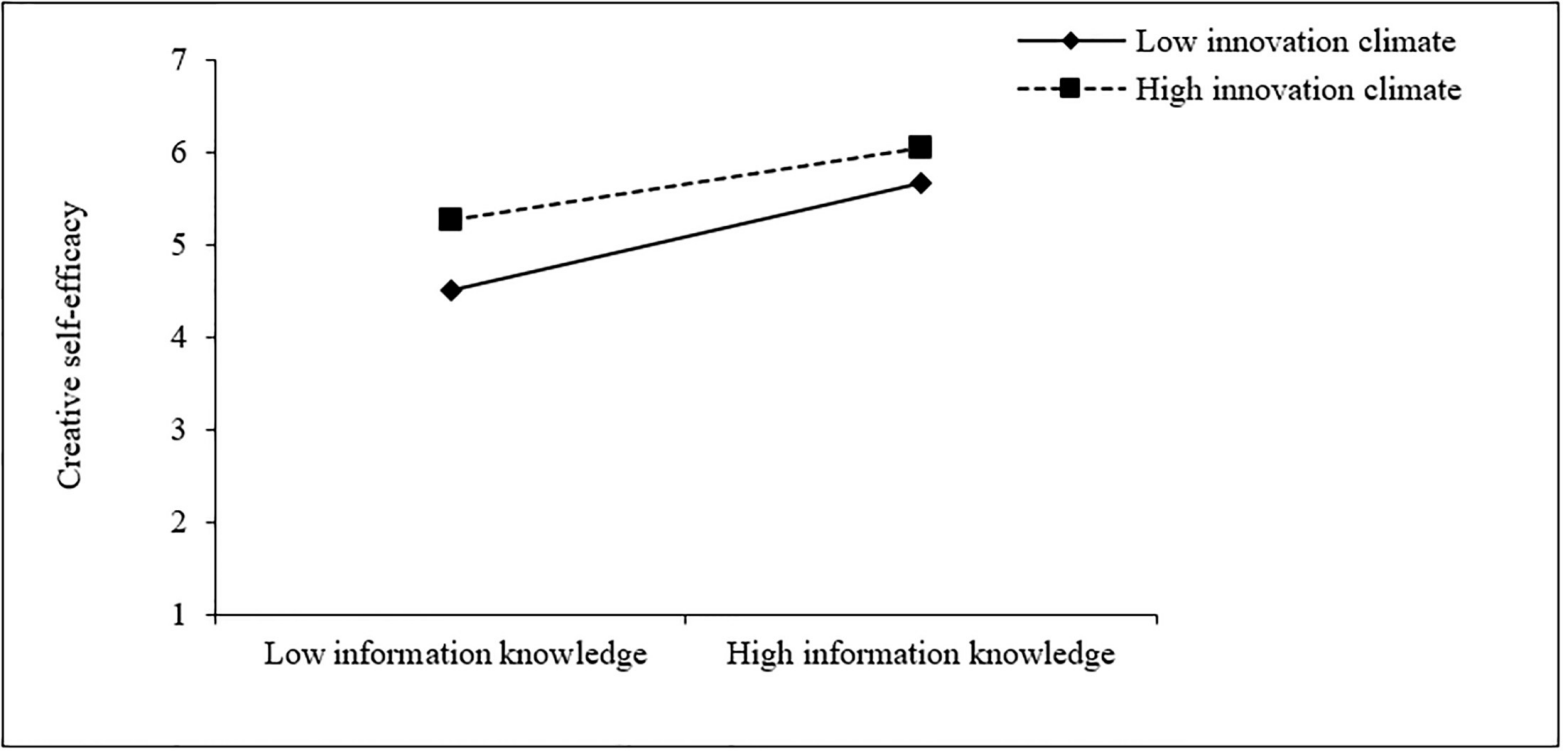
The moderating effect of college innovation climate on the relationship between teachers’ information knowledge and creative self-efficacy.

Similarly, according to Figs 3 and 4, when the college innovation climate is low (M-1SD), teachers’ information awareness (simple slope = 0.60, t = 7.54, p < 0.001), information knowledge (simple slope = 0.85, t = 9.26, p < 0.001) also has a stronger positive effect on their creative self-efficacy than that in the case of higher innovation climate (M+1SD) (simple slope = 0.28, t = 2.77, p < 0.01, simple slope = 0.56, t = 6.77, p < 0.001, respectively). This indicates that when the innovation climate in colleges and universities is poor, teachers’ information awareness and information knowledge have a greater effect on their creative self-efficacy, which further supports H3a and H3b.

The study also used Model 7 of the Process V3.4 macro program to examine the mediating effect of creative self-efficacy between information literacy (and its dimensions) and innovative teaching behavior under different levels of college innovation climate. The results show (see Table 6) that when the college innovation climate is low and high, there is a significant difference in the indirect effect of information literacy on innovative teaching behavior through creative self-efficacy (difference value is -0.07, 95% confidence interval does not include 0). Therefore, it can be concluded that the college innovation climate moderates the mediating effect of creative self-efficacy between teachers’ information literacy and innovative teaching behavior. That is, H4 is supported. The moderated mediation index is -0.05, and the 95% confidence interval does not contain 0, further verifying H4.

**Table 6:** The moderated mediation effect.

Effect	IL → CSE → ITB			IA → CSE → ITB			IK → CSE → ITB		
	β	Boot SE	95% CI	β	Boot SE	95% CI	β	Boot SE	95% CI
Low CIC	0.283	0.055	[0.185, 0.403]	0.245	0.041	[0.172, 0.333]	0.294	0.046	[0.204, 0.387]
High CIC	0.209	0.061	[0.111, 0.347]	0.110	0.051	[0.024, 0.227]	0.192	0.045	[0.164, 0.322]
Difference	-0.074	0.031	[-0.124, -0.002]	-0.135	0.039	[-0.202, -0.048]	-0.103	0.041	[-0.174, -0.007]
Index	-0.054	0.023	[-0.090, -0.002]	-0.098	0.028	[-0.146, -0.035]	-0.074	0.030	[-0.126, -0.005]

Note: 95% CI is 95% confidence interval.

As for the three dimensions of information literacy, when the college innovation climate is low and high, the indirect effects of information awareness and information knowledge on innovative teaching behavior through creative self-efficacy are also significant (the difference values are -0.14 and -0.10 respectively, and the 95% confidence intervals are [-0.202, -0.048] and [-0.174, -0.007] respectively); The moderated mediation index are -0.10 and -0.07 respectively, none of the 95% confidence intervals contain 0, which means that H4a and H4b are supported. The result of Process V3.4 macro program also shows that the indirect effect difference of information capability on innovative teaching behavior through innovation self-efficacy is -0.04 when the college innovation climate is low and high, and 95% confidence interval ([-0.093, 0.032]) contains 0; Besides, the 95% confidence interval ([-0.071, 0.025]) of the moderated mediation index also contains 0, indicating that H4c is not supported.

## 5 Conclusions and discussion

### 5.1 Discussion

The study found the significant positive impact of college teachers’ information literacy on their innovative teaching behavior, which was highly consistent with that of former scholars [1,9]. This could be because college teachers with a high level of information literacy have the high sensitivity and consciousness to information and new information technologies, have a good grasp of the knowledge related to new teaching technologies, and are capable of searching, integrating and utilizing information related to teaching innovation, and applying these technologies, knowledge and information into teaching practice [21]. Among the three dimensions of information literacy, information knowledge and information capability have significant effects on innovative teaching behavior. So, it is necessary to improve the information literacy of college teachers, especially improve their information knowledge and information capability level. This requires college teachers to take the initiative to acquire and understand the latest knowledge in their professional fields, timely learn and master new information technologies such as big data, cloud computing and artificial intelligence, and actively try to deeply integrate these new knowledge and new technologies into course teaching. For example, college teachers can flexibly use online teaching platforms to carry out innovation in teaching models such as micro-class and flipped classroom, and use big data technology to grasp students’ learning interests and learning situations, and then innovate teaching content and teaching methods.

The research also verified the mediating role of creative self-efficacy between college teachers’ information literacy (information knowledge and information capability) and their innovative teaching behavior. This shows that while the higher the level of information literacy (information knowledge and capability) of college teachers, the stronger their ability to understand and apply new technologies to carry out teaching innovation, thus the more confident they are to overcome the difficulties and challenges encountered in the process of teaching innovation and achieve the expected teaching innovation effect, and the stronger their motivation and tendency to carry out teaching innovation [5]. Therefore, on the one hand, college teachers can use various channels actively to improve their information knowledge and information ability so as to enhance their information literacy level; on the other hand, colleges and universities should also give timely affirmation and encouragement to teachers’ teaching innovation to make them feel fully recognized and respected, and thus enhance their sense of creative self-efficacy and stimulate their innovative teaching behavior.

As for the moderation effect, it was found that innovation climate in colleges and universities significantly moderated the positive relationship between information literacy (information awareness and information knowledge) and creative self-efficacy, and then moderated the mediating effect of creative self-efficacy between information literacy (information awareness and information knowledge) and innovative teaching behavior. In other words, under the condition of low innovation climate, the effect of information literacy (information knowledge) on innovative teaching behavior through teacher’ creative self-efficacy is stronger; Vice versa. It is worth noting that information awareness itself cannot affect innovative teaching behavior through creative self-efficacy. However, in the context of poor college innovation climate, teachers with strong information awareness have stronger sense of creative self-efficacy and tend to carry out more innovative teaching activities. This further illustrates the key significance of college innovation climate to their innovative teaching psychology and behavior in the information age. So, colleges and universities should create a good innovation climate, with strong support and rewards to significantly enhance teachers’ positive initiative to carry out teaching innovation. In this regard, colleges and universities should increase investment, gradually improve the information and intelligent construction of teaching equipment, and provide hardware support for teachers to carry out teaching innovation; At the same time, it is also necessary to introduce and improve a set of mechanisms to support teaching innovation, including teaching innovation awards, re-education training, and teachers’ teaching and academic assessment to create an innovative climate that advocates reform and permits errors, encourage teachers to innovate boldly and practice actively, with expectation to build and form a number of first-class undergraduate majors at the world level.

### 5.2 Conclusions

This research aimed to explore the mechanism and boundary conditions of college teachers’ information literacy and its dimensions affecting their innovative teaching behavior based on the theory of social cognition and theory of behavioral change. To achieve the first goal of the study, the research verified the effect of college teachers’ information literacy on their innovative teaching behavior firstly, and further examined how do the three dimensions of information literacy affect innovative teaching behavior. 280 valid data showed that college teachers’ information literacy and its dimensions of information knowledge and information capability had a significant positive effect on their innovative teaching behavior, the influence of information awareness on innovative teaching behavior is not significant. Moreover, there are differences in the impact of the three dimensions of information literacy on innovative teaching behavior. Among the three dimensions, the influence of information capability on innovative teaching behavior is the greatest.

Next, the study was also interested in the action mechanism of college teachers’ information literacy (and its dimensions) on their innovative teaching behavior, so the researcher examined the mediating role of creative self-efficacy. It was found that creative self-efficacy played a significant mediating role between information literacy (and its information knowledge and information capability dimensions) and innovative teaching behavior, but there was no evidence in the study for supporting the mediating effect of creative self-efficacy between information awareness and innovative teaching behavior.

The third purpose of the study was to investigate the moderating effect and the moderated mediating effect of college innovation climate to explore the boundary conditions of the action mechanism. The results showed that college innovation climate significantly moderated the positive relationship between information literacy (information awareness and information knowledge) and creative self-efficacy, and significantly moderated the mediating effect of creative self-efficacy between information literacy (information awareness and information knowledge) and innovative teaching behavior. However, the study found no supports for the moderating effect of college innovation climate on the relationship between information capability and creative self-efficacy, neither for the moderating effect on the mediating role of creative self-efficacy between information capability and innovative teaching behavior.

On the whole, the possible novelties and theoretical contributions of the study are as follows: Firstly, on the basis of verifying the influence of information literacy on teaching innovation, the study further examined the effect of its three dimensions on innovative teaching behavior, and obtained some new viewpoints, including different dimensions of information literacy had different impacts on teaching innovation; among three dimension, information capability had the most powerful impact on innovative teaching behavior, which can strongly explain the causes of college teachers’ teaching innovation under the background of informatization, and can further enrich the existing research on the factors influencing innovative teaching behavior. Second, As mentioned above, there is no research on the mechanism of information literacy affecting innovative teaching behavior. The study examined the mediating role of creative self-efficacy between information literacy (its dimensions) and innovative teaching behavior, which not only revealed the “black box” in which college teachers’ information literacy affects teaching innovation, but also positively responded to some scholars’ call for strengthening the exploration of the mechanism of innovative teaching behavior influenced by various influential factors [22]. Finally, the research innovatively explored the moderating effect and the moderated mediating effect of college innovation climate, the findings of which can not only explain teaching innovation more comprehensively by combining internal and external factors, but also help to enrich the research on the situational factors of the mechanism of college teachers’ innovative teaching behavior under the background of education informatization.

### 5.3 Limitations and future research

There are still some research limitations. The sample data collected in the study is mainly concentrated in some universities from central areas, which brings some limitations in regional selection and sample number. Future research can cover universities in eastern and western regions, collect more representative sample data, track the influence of college teachers’ information literacy in different regions on their innovative teaching behavior, and improve the universality of the research conclusions. Besides, this study only uses the questionnaire method to explore the influence of college teachers’ information literacy on their innovative teaching behavior, in which the research method is relatively simple, and the research conclusions may also be biased. Future studies can be combined with semi-structured in-depth interviews to further explore the influential factors and mechanisms of college teachers’ innovative behavior in information-based teaching, and expand the research conclusions of this research.

## Supporting information

S1 Dataset (SAV)
